# Expression of MxA in esophageal cancer cell lines can influence sensitivity to chemotherapeutic agents but this does not require apoptosis

**DOI:** 10.1002/cam4.70173

**Published:** 2024-09-16

**Authors:** R. M. Hayes, T. R. O'Donovan, S. L. McKenna

**Affiliations:** ^1^ Cancer Research @UCC College of Medicine and Health, University College Cork Cork Ireland

**Keywords:** apoptosis, drug resistance, esophageal cancer, MX1, MxA

## Abstract

Esophageal cancer is a poor prognosis cancer characterized by intrinsic or acquired resistance to chemotherapeutic agents. The primary determinants of treatment failure are unknown. Expression of an anti‐viral protein, myxovirus resistance protein A (MxA) is de‐regulated in many cancers, including esophageal cancer, and its activity has been linked to apoptosis. This study has assessed whether MxA expression can influence the response of esophageal cancer cells to the chemotherapeutic agents 5‐fluorouracil (5‐FU) or oxaliplatin. MxA protein was differentially expressed in a panel of five esophageal cancer cell lines. KYSE450 and KYSE140 cells did not express MxA and were apoptosis incompetent. FLO‐1, KYSE270, and OE21 cells expressed MxA, were more drug‐sensitive and were apoptosis competent. MxA was artificially overexpressed in cell lines with no endogenous expression (KYSE450 and KYSE140). This increased the resistance of KYSE450 but not KYSE140 cells. Both cell lines remained apoptosis incompetent. We then evaluated siRNA knockdown of MxA in FLO‐1 cells and CRISPR knockout in OE21 cells. Knockdown of MxA significantly increased drug sensitivity and caspase‐3 activation in FLO‐1 cells. OE21‐*MX1* ^KO^ cells were also more drug‐sensitive, but in contrast to FLO‐1 cells, caspase‐3 activation was reduced. Collectively these data indicate that MxA can promote resistance to chemotherapy, but this does not always correspond with effects on apoptosis. Effects on apoptosis are cell line specific, suggesting that other co‐operating pathways determine the overall impact of MxA. Importantly, in cancer cells that overexpress the protein, drug sensitivity can be improved by interfering with MxA.

## Graphical abstract

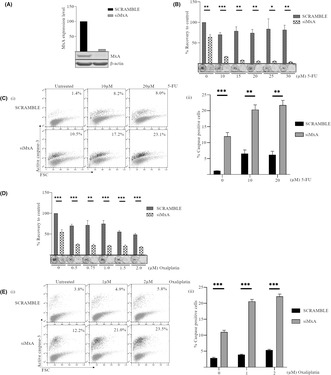

## 1 INTRODUCTION

Esophageal cancer is the seventh most common cancer worldwide. It is the sixth most common cause of cancer death. Although the 5‐year survival rate has improved over the last 20 years, it remains below 20%.^1^, ^2^ A key factor in the poor prognosis associated with esophageal cancer is the development of drug resistance and recurrence of disease. Improved activation of programmed cell death (PCD) pathways within cancer cells following treatment with chemotherapeutics is one way to improve treatment outcomes.^3^ However, resistance to PCD is one of the key hallmarks of cancer^4^ and this is a limitation that is difficult to overcome.

We have previously explored differences in gene expression in esophageal cancer cell lines that respond differently to standard chemotherapeutics. This study highlighted a network of interacting, interferon‐stimulated genes (ISG's), that are differentially expressed between drug‐sensitive and drug‐resistant cell lines.^5^ One of these genes, *MX1* was found to be upregulated in more sensitive, apoptosis‐competent cells, but absent from the more resistant cell lines. The protein product of the *MX1* gene is MxA, a 78 kDa dynamin‐like GTPase. It was originally identified as a factor that conveyed immunity to many types of myxovirus in an inbred strain of mice.^6^ MxA has anti‐viral activity against a range of single and double‐stranded RNA viruses and some DNA viruses (reviewed in^7^). It has also been more recently associated with the severity of responses to COVID‐19.^8^, ^9^ Expression of MxA is regulated by type I and type III interferons, which can increase the amount of MxA within the cell by greater than 200‐fold.^7^ The molecular mechanisms by which MxA achieves its broad range of anti‐viral activity are the subject of intensive investigation but have not yet been fully elucidated.

Several studies have now suggested that MxA may play an important role in cancer. Analysis of the human *MX1* gene in the Catalog of Somatic Mutations in Cancer (COSMIC) database identified 122 mutations across many common cancers. These included truncations from termination codons or frameshift mutations and 22 unique single amino‐acid mutations predicted to affect key functional domains in the protein.^10^ The consequence of these mutations for cancer biology is currently unknown.

A study in breast carcinoma indicated that expression of a type I IFN‐related signature, including MxA, is a positive indicator for patients who might benefit from anthracycline‐based chemotherapy.^11^ MxA expression was also found to be higher in triple‐negative breast cancer, compared to other subtypes. In these patients, high MxA levels and abundant tumor‐infiltrating lymphocytes were found to be independent prognostic factors for disease‐free survival.^12^ Conversely, another study assessed MxA at the protein level using breast cancer tissue microarrays and mRNA expression from publicly available data sets. High MxA protein expression or mRNA expression was associated with more aggressive tumors and shorter survival.^13^

Analysis of prostate cancer data sets in cBioPortal indicated that deletions were the most common genetic alteration in *MX1* and were found in over 13% of patients.^14^ Bioinformatic analysis of a prostate cancer data set with extended clinical follow‐up (GSE70770),^15^ indicated that patients with lower expression of *MX1* transcript had worse relapse‐free survival.^14^ MxA is also frequently deleted in prostate cancer as a consequence of the TMPRSS2‐ERG fusion, which is linked to more aggressive and invasive prostate cancer.^16^ Functional studies have been undertaken in several prostate cancer cell lines. Loss of MxA has been associated with increased proliferation, migration, and reduced apoptosis in response to the chemotherapeutic drug docetaxel.^17^

Several groups have analyzed genomic and gene expression data from esophageal cancer patients,^18^, ^19^ to identify molecular subtypes that might be therapeutically relevant for patient stratification or to identify driver genes that might influence therapeutic development. MxA was not highlighted in such studies (to our knowledge). However, a DNA Damage Immune Response signature (DDIR assay), in which MxA is a high‐ranking gene, was applied to esophageal cancer datasets. This was found to be predictive of response and survival benefit following neoadjuvant DNA‐damaging chemotherapy.^20^ A recent proteomics analysis of 124 squamous esophageal tumor samples identified MxA as part of a network of ISGs which are more highly expressed in tumor samples compared to non‐tumor adjacent tissue. They also noted that the high expression of these proteins did not correspond to gene expression data, highlighting the need to examine the expression of these immune‐related effectors at the protein level.^21^

As induction of PCD is a well‐documented response of cells in multicellular organisms to viral infection,^22^, ^23^ several groups have investigated whether MxA can influence apoptosis or other forms of PCD. Expression of MxA was reported to promote the apoptotic pathway in influenza virus‐infected cells exposed to anti‐FAS antibody or irradiation.^24^ In contrast, a study evaluating apoptosis induction following vesicular stomatitis virus (VSV) infection of pancreatic carcinoma cells, found that cell lines that were deficient in MxA had high levels of caspase‐3 activation.^25^

Increased cell death was also observed following overexpression of MxA in Swiss mouse 3T3 cells, following cycloheximide treatment, irradiation, or viral infection.^26^ The increase in cell death mediated by viral infection was not associated with caspase activation. Increased apoptosis was reported following MxA overexpression in HEP3B cells and apoptosis was further enhanced in response to mitomycin C treatment.^27^ MxA has also been reported to induce a caspase‐independent ER stress response following treatment with the ER‐stress inducer tunicamycin.^28^ Induction of ER stress following treatment with thapsigargin also resulted in increased *MX1* mRNA levels, and this was associated with increased apoptosis and autophagy in PC3 cells.^14^ Therefore, MxA can influence cell death in various cell types and in response to many different stimuli.

In this study, we have investigated whether expression of MxA can influence the response of esophageal cancer cell lines to chemotherapeutic drugs (5‐fluorouracil and oxaliplatin) and assessed whether this response is associated with modulation of apoptosis.

## 2 MATERIALS AND METHODS

### 2.1 Cell culture/reagents

Human esophageal cancer cell lines OE21 and FLO‐1 were obtained from the European Collection of Cell Cultures. KYSE450, KYSE140, and KYSE270 cell lines were from DSMZ (Deutsche Sammlung von Mikroorganismen und Zellkulturen GmbH). OE21, KYSE450, KYSE140, and KYSE270 cells were derived from squamous cell carcinoma, while FLO‐1 cells were derived from adenocarcinoma. OE21, FLO‐1, and KYSE140 cells were grown in RPMI 1640 medium (Merck, R8758). KYSE450 and KYSE270 cells were maintained in 50:50 RPMI/F12 HAMS medium (Merck, N6658), all supplemented with 10% fetal bovine serum (Merck, F7524) and 1% Penicillin/Streptomycin (Gibco‐BRL, 15070–063) and grown at 37°C, 5% CO2. 5‐fluorouracil (5‐FU) (F6627) and oxaliplatin (O9512) were purchased from Merck. Recombinant interferon α‐2b (Intron A) was provided by Schering Corporation (NDC‐0085) and used at 10,000 IU, or from ENZO Life Sciences (NZ‐PRT192‐0010) and used at 200 pg–100 ng.

### 2.2 Transfections

To achieve overexpression of MxA, cells were reverse transfected with the following plasmids: an empty vector pcDNA3.1‐HA (Addgene plasmid # 128034), MxA or MxA_T103A (both gifts from Otto Haller, Institute of Virology, University Medical Center Freiburg), using TurboFect (Thermo, R0531) according to manufacturer's instructions. To achieve knockdown of MxA, cells were reverse transfected with short‐interfering RNA (siRNA) directed at MxA (IDT, hs.Ri.MX1.13.1) or scrambled negative control (IDT, 51‐01‐19‐09) using RNAiMAX (Invitrogen, Lipofectamine RNAiMAX Transfection Reagent #13778150).

### 2.3 Western blotting

Whole‐cell protein lysates were prepared by lysing cells in modified RIPA buffer (50 mM Tris–HCl (pH 7.4), 150 mM NaCl, 0.25% sodium deoxycholate, 1% Igepal, 1 mM EDTA, 1x Pefabloc, 1 × protease inhibitor cocktail (Roche, cOmplete mini EDTA‐free), 1 mM Na3VO4, 1 mM NaF). Protein samples (60 μg) were separated on NuPAGE 4%–12% Bis‐Tris gels (Invitrogen, NP0322) and electrophoretically transferred onto PVDF membrane using the BioRad Trans‐Blot Turbo gel transfer system (BioRad, 1704150). Membranes were incubated with anti‐MxA antibody (Proteintech, 13750‐1‐AP, 1:1000) overnight at 4°C followed by incubation with relevant IR‐Dye conjugated secondary antibodies (Li‐Cor) and imaged on the Odyssey IR imaging system (Li‐Cor). Anti‐β‐actin antibody (Merck A5441, 1:10,000) was used as a loading control.

### 2.4 Colony formation assay

Cells were treated with chemotherapeutic drugs 5‐FU and oxaliplatin for 24 or 48 h. In drug‐free media, 1500 viable cells were reseeded in a well of a six‐well plate, in triplicate, and allowed to recover for up to 14 days. Colonies were fixed in 99% methanol and stained with Rapi‐Diff II. Colony number and density were analyzed using the Odyssey infrared imaging system (Li‐Cor). Results are presented as integrated intensity ± standard error of the mean (SEM).

### 2.5 Caspase‐3 assay

Cells were fixed with a final concentration of 2% paraformaldehyde (PFA) and permeabilized with IFA‐TX buffer (0.1% Triton X‐100, 0.1% sodium azide, 10 mM HEPES, 4% FCS, 150 mM NaCl). Cells were incubated with active caspase‐3 antibody (BD Biosciences, 559565) for 1 h at 4°C followed by Alexa fluor‐488 secondary antibody (ThermoFisher Scientific, A11034). Samples were analyzed using the BD LSR II flow cytometer and BD FACS Diva acquisition and analysis software. Data are presented graphically as percentage caspase positive cells ± SEM.

### 2.6 Generation of OE21 knockout cell line with CRISPR‐Cas9

Guide RNA sequences for CRISPR‐Cas9 were designed using IDT CRISPR design software. The guide RNA that proved most successful was targeted at exon 10 of the coding sequence of *MX1*. Purified Cas9 nuclease (Alt‐R® S.p. Cas9 Nuclease V3, 1081058), tracrRNA (Alt‐R® CRISPR‐Cas9 tracrRNA, 1072532) and guide RNA (Hs.Cas9.MX1.1.AA, sequence: /AlTR1/rArArUrCrUrUrGrArCrGrArArGrCrCrUrGrArUrCrGrUrUrUrUrArGrArGrCrUrArUrGrCrU/AlTR2/) were all purchased from IDT (IDT DNA, Leuven, Belgium). The complimentary RNA molecules were annealed and incubated with the Cas9 nuclease for 5 min at room temperature to form the RNP complex. The RNP complex was reverse transfected in a 96‐well plate using RNAiMAX (Invitrogen, Lipofectamine RNAiMAX Transfection Reagent #13778150) according to manufacturer's instructions. Forty‐eight hours after transfection, clones were isolated by serial dilution and knockout was confirmed by whole transcriptome mRNA sequencing and western blotting.

### 2.7 Examination of morphology

Control and drug‐treated cells were cytospun onto glass slides and stained with Rapi‐Diff I and II (BAPROD1, Braidwood Laboratories). Cytospin images are representative of at least three independent experiments. Images were captured using an Olympus BX51 microscope with an Olympus DP74 digital microscope camera at 400X magnification.

### 2.8 Statistical analysis

All statistical analysis was conducted using GraphPad Prism version 8 software. Comparisons between groups were assessed using Student's *t*‐test. The *p* value was considered statistically significant when *p < 0.05, **p < 0.01, ***p < 0.001. Exact *p* values are given where the value is greater than or equal to 0.001. *p* values less than 0.001 are reported as *p* < 0.001.

## 3 RESULTS

### 3.1 Esophageal cancer cell lines have variable expression of MxA and exhibit a range of sensitivity to oxaliplatin and 5‐fluorouracil

**FIGURE 1:**
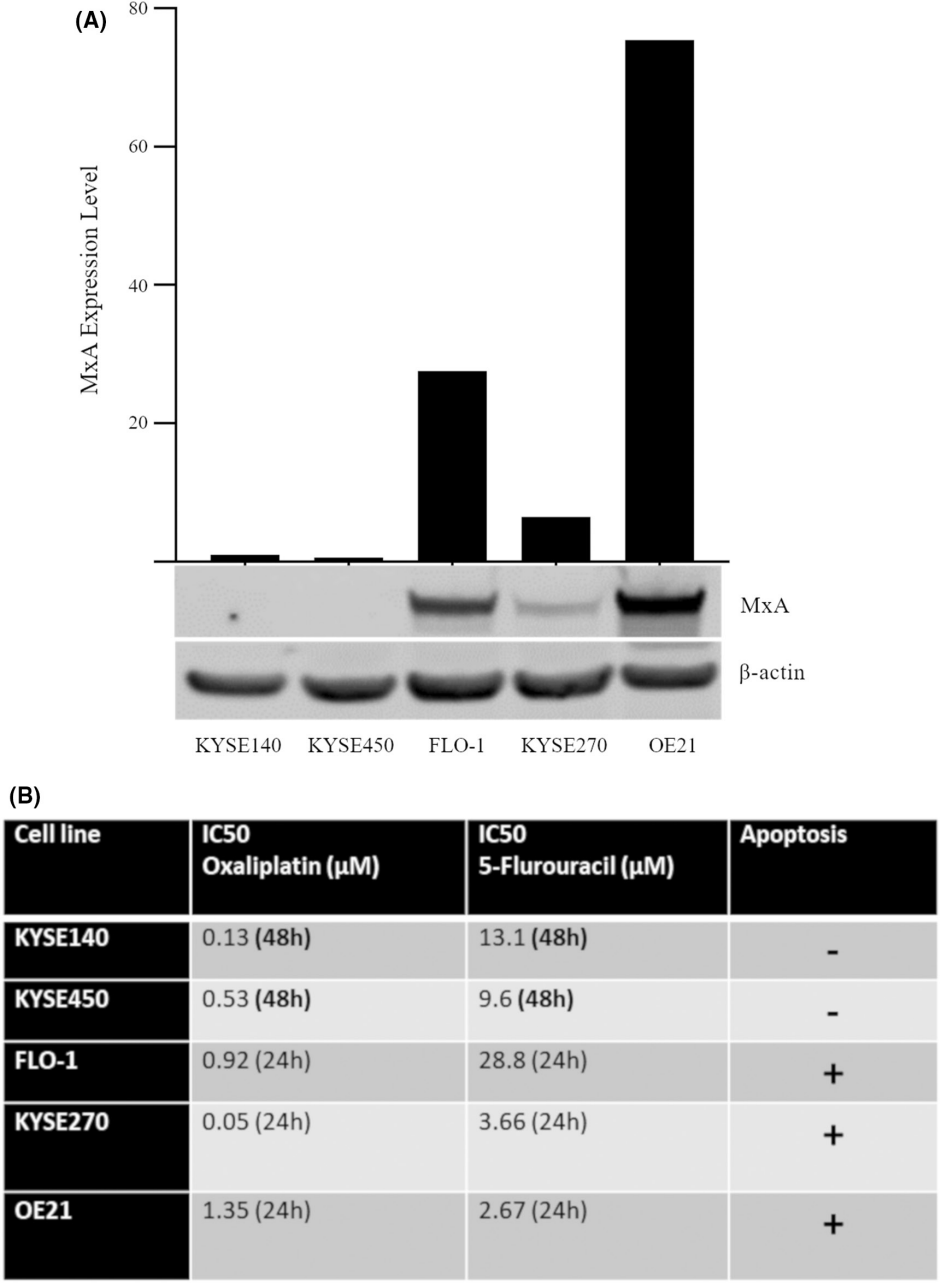
MxA protein expression and apoptosis competency in a panel of esophageal cancer cell lines. (A) Western blot analysis of endogenous expression of MxA in five esophageal cancer cell lines. MxA expression was quantified with the Odyssey infrared imaging system (LiCor) and normalized to β‐actin, with normalized integrated intensities graphed above. Western blot shown is representative of at least three independent experiments. (B) Table summarizing the IC50 and apoptosis competency of a panel of esophageal cancer cell lines in response to 5‐fluorouracil (5‐FU) or oxaliplatin treatment for either 24 or 48 h. Apoptosis was defined by both morphology and caspase‐3 activation (detailed in the supplementary data for each cell line) (*n* = 3).

Endogenous protein expression of MxA was assessed in a panel of esophageal cancer cell lines (KYSE140, KYSE450, FLO‐1, KYSE270, and OE21) (Figure 1A). KYSE450 and KYSE140 cells did not express MxA, whereas FLO‐1, KYSE270, and OE21 cells expressed MxA at detectable, but variable levels. The panel of cell lines was then assessed for their sensitivity to the chemotherapeutic drugs 5‐FU and oxaliplatin.

FLO‐1, OE21, and KYSE270 cells were more sensitive to both chemotherapeutic agents, as toxicity was evident within 24 h. This group of cell lines also induced caspase‐3‐mediated apoptosis in response to both drugs. (Summarized in Figure 1B and all associated data is shown in Figure S1A–C).

KYSE450 and KYSE140 cells were more resistant to both 5‐FU and oxaliplatin. Evaluation of clonogenic recovery and examination of morphologies following 24 h of drug treatment found no cytotoxic effect at this time point (*data not shown*). Toxicity was only evident after 48 h of treatment, and apoptotic morphology and activation of caspase‐3 were rare at all time points (Figure 1B and Figure S1D–E).

### 3.2 Overexpression of MxA enhanced resistance of KYSE450 cells to 5‐fluorouracil and does not influence apoptosis competency in the absence or presence of interferon‐α

**FIGURE 2:**
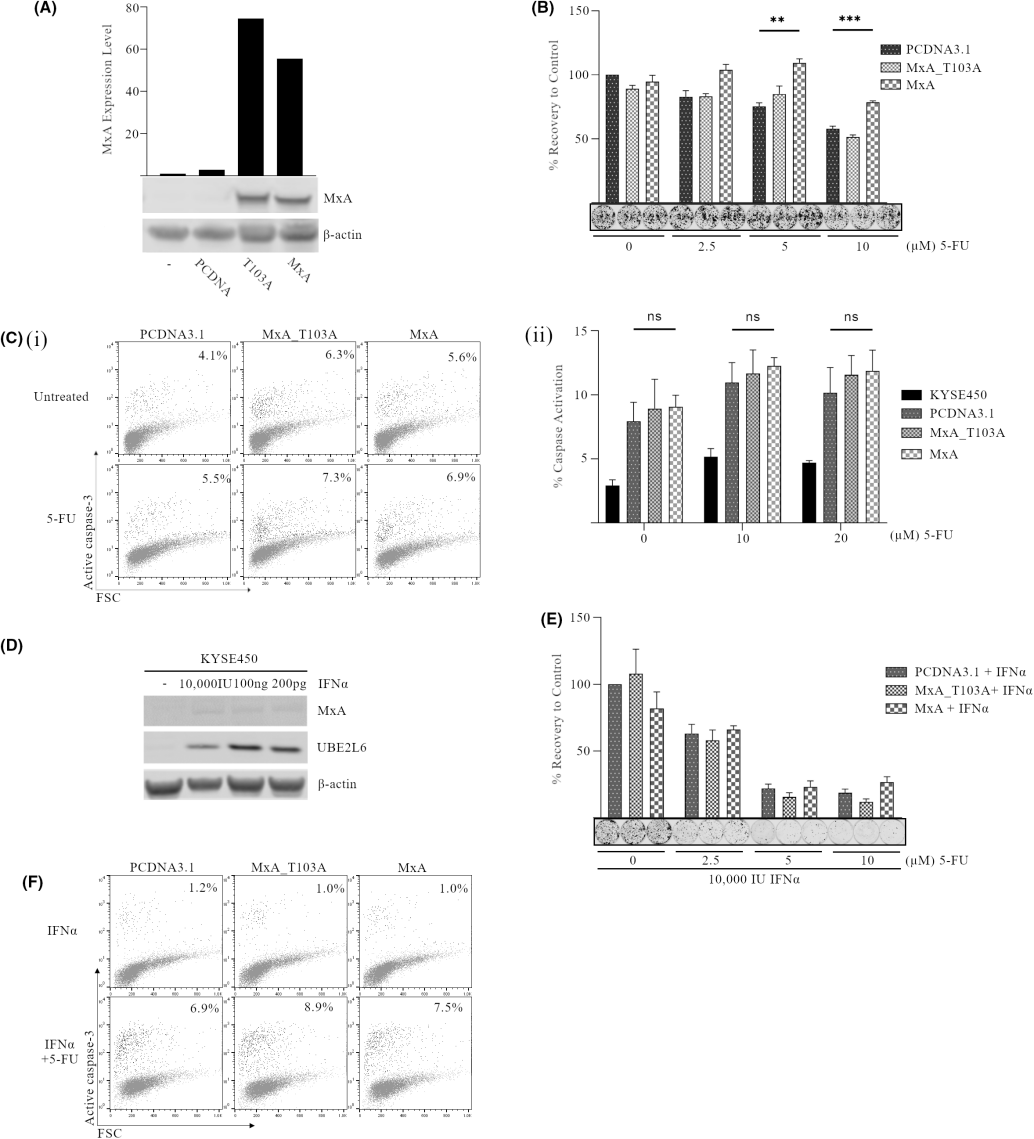
Overexpression of MxA enhanced resistance of KYSE450 cells to 5‐Fluorouracil (5‐FU) but does not influence apoptosis competency in the absence or presence of interferon‐α (IFN‐α). KYSE450 cells transiently expressing MxA, MxA_T103A mutant, or empty vector control (PCDNA3.1) were treated with 5‐FU (2.5–10 μM) in the absence (A–C) or presence of interferon‐α (IFN‐α) (D‐F) for 48 h. (A and D) Expression levels of MxA and UBE2L6 were assessed by western blot in untransfected KYSE450 cells and cells transfected with MxA, MxA‐T103A, or PCDNA3.1. β‐Actin was used as a loading control. Western blot shown is representative of at least three independent experiments. (B and E) Following drug removal, a colony formation assay was conducted to determine the ability of KYSE450 cells expressing MxA or MxA‐T103A to recover, relative to empty vector controls (PCDNA3.1). Colonies were stained using Rapi‐diff and quantified using the Odyssey infrared imaging system. Triplicate data is presented in the graph as integrated intensity ± SEM (**p < 0.01, ***p < 0.001). (C and F) KYSE450 cells expressing MxA, MxA‐T103A or empty vector controls were treated with 5‐FU (10 and 20 μM) for 48 h and were analyzed for levels of active caspase‐3 by flow cytometry. Data from three independent experiments is presented as % caspase positive cells.

The effect of MxA overexpression on chemosensitivity in the more resistant KYSE450 cell line was initially evaluated. MxA or a GTPase‐inactive MxA_T103A mutant were transiently overexpressed. An empty vector, PCDNA3.1, was used as a control throughout. MxA expression was confirmed by western blot (Figure 2A). Cells overexpressing MxA, MxA_T103A, or empty vector were treated with a range of concentrations of 5‐FU (2.5‐10 μM) for 48 h. To assess the ability of cells to recover and form colonies following drug treatment, equal numbers of viable cells were reseeded and incubated for up to 14 days in drug‐free media. KYSE450 cells overexpressing MxA showed a modest but significant increase in recovery from 5‐FU treatment at 5 μM (*p* = 0.0012) and 10 μM (*p* < 0.001), compared to the empty vector controls (Figure 2B). There was also a significant difference in recovery between MxA and the GTPase inactive mutant (MxA_T103A) at 2.5, 5, and 10 μM 5‐FU. Thus, GTPase activity may be required for the increased survival associated with MxA overexpression after 5‐FU treatment.

Levels of active caspase‐3 were also assessed following treatment with 5‐FU (10 and 20 μM) for 48 h. KYSE450 cells typically show very low levels of active caspase‐3 (<6% Figure S1D (ii)). Transfection with plasmid DNA induced minimal caspase‐3 activation in all transfected cells, consistent with the presence of foreign DNA. However, there was no significant difference in the levels of active caspase‐3 in the control plasmid and MxA transfected cells (Figure 2C i and ii). These data indicate that overexpression of MxA in KYSE450 cells can marginally improve recovery following drug treatment, but it does not influence caspase‐3‐mediated apoptosis. Similar data were obtained following treatment with oxaliplatin (Figure S2A–C). A modest increase in recovery with both MxA plasmids was observed, with no difference in apoptosis induction.

MxA is an interferon‐inducible gene and may require the expression of other IFN‐inducible genes for its full activity.^29^ We therefore repeated the overexpression of MxA in the presence of IFN‐α (Figure 2D–F). Interestingly, MxA itself was not induced in response to IFN‐α treatment (Figure 2D), despite MxA being the most common marker of Type I IFN treatment. However, other interferon‐stimulated genes, such as UBE2L6 (Figure 2D), and USP18 (not shown), were upregulated. This indicates that the type I interferon pathway is responsive in these cells. KYSE450 cells were again transfected with plasmids overexpressing MxA, MxA_T103A, or empty vector control and treated with 5‐FU (2.5–10 μM) in the presence of IFN‐α (10,000 IU). Cells exposed to IFN‐α showed increased sensitivity to 5‐FU treatment across all concentrations of 5‐FU examined (Figure 2E). For example, cells transfected with PCDNA3.1 and treated with 5 μM 5‐FU alone showed 85% colony recovery (Figure 2B). In contrast, only 25% recovery was evident with the addition of IFN‐α, indicating an enhanced cytotoxic effect of 5‐FU in the presence of IFN‐α. However, overexpression of MxA had no additional effect on sensitivity (Figure 2E). Activation of caspase‐3 was marginally increased in the presence of IFN‐α in all cells, irrespective of the plasmid introduced (Figure 2F). Therefore, MxA overexpression in the presence of IFN‐α does not affect drug sensitivity or caspase‐3 activation in KYSE450 cells.

This work was repeated in the second non‐expressing cell line, KYSE140. MxA overexpression had no effect on sensitivity to 5‐FU or apoptosis induction in the presence or absence of IFN‐α (Figure S3A–F).

### 3.3 siRNA knockdown of MxA increases the sensitivity of FLO‐1 cells to 5‐fluorouracil and oxaliplatin and is associated with enhanced apoptosis

**FIGURE 3:**
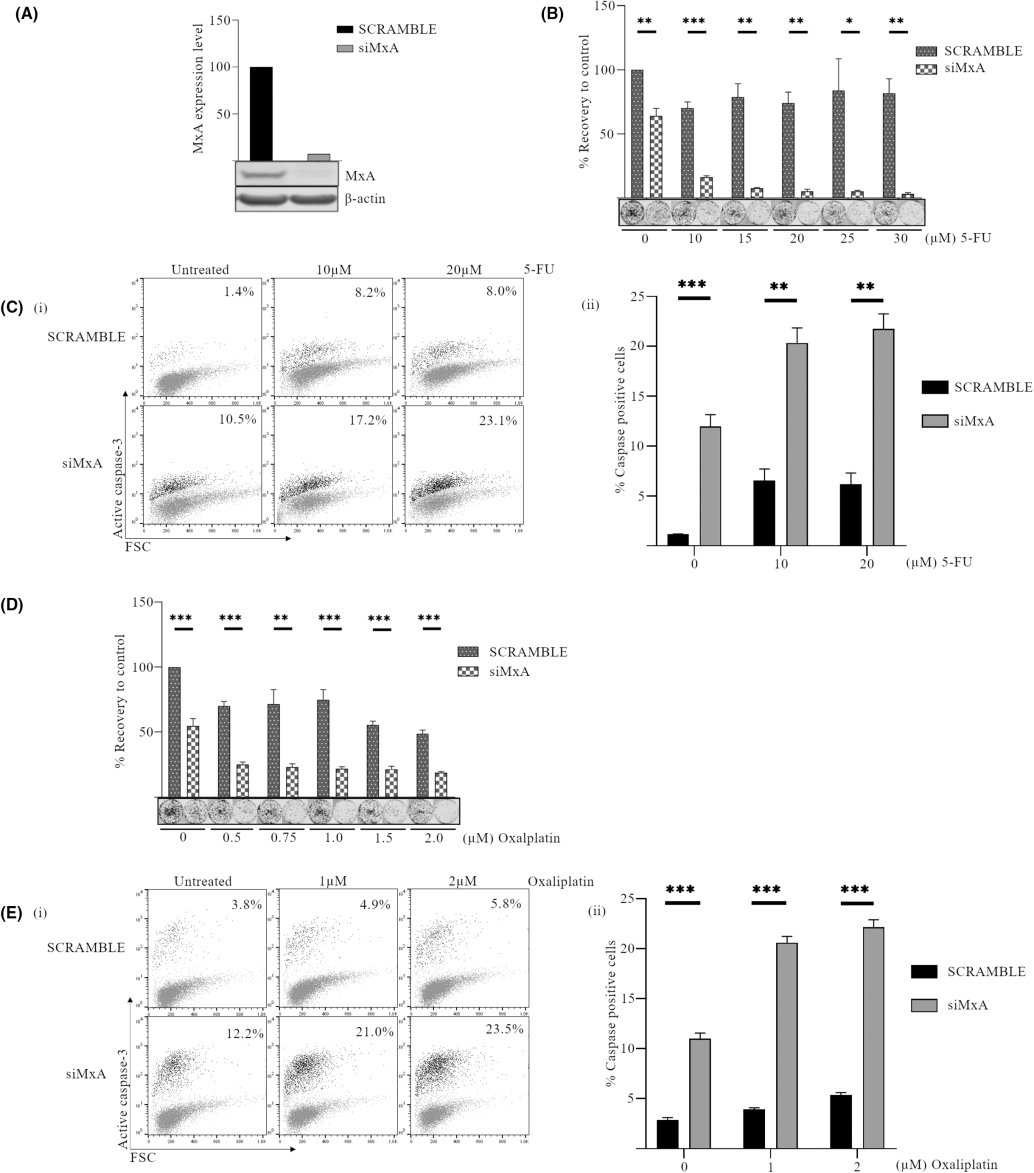
siRNA‐mediated knockdown of MxA increases sensitivity of FLO‐1 cells to 5‐fluorouracil (5‐FU) and oxaliplatin and is associated with enhanced caspase‐3 activation. FLO‐1 cells were transfected with siRNA directed at MxA (siMxA) or scramble control (Scr) and treated for 24 h with either 5‐FU (10–30 μM) or oxaliplatin (0.5–2.0 μM) (A) Knockdown of MxA was confirmed by western blot, β‐actin was used as a loading control and all bands were quantified using the Odyssey infrared imaging system. (B and D) Following relevant drug treatment, FLO‐1 cells without or with MxA were reseeded in drug‐free medium to examine their ability to recover and form colonies. Colonies were stained using Rapi‐diff and quantified using the Odyssey infrared imaging system. Triplicate data are presented as integrated intensity ± SEM. (C and E) Levels of active caspase‐3 were assessed by flow cytometry. Representative dot plots are shown (i) with mean percentage caspase‐positive cells for three independent experiments graphed in (ii) ± SEM (*n* = 3). (*p < 0.05, **p < 0.01, ***p < 0.001).

We also evaluated the effects of siRNA‐mediated knockdown of MxA in the endogenously expressing FLO‐1 cell line. Knockdown was confirmed by western blot (Figure 3A). Cells transfected with scramble siRNA (Scr), or siRNA directed at MxA (siMxA) were treated with a range of concentrations of 5‐FU (10–30 μM) for 24 h. Following treatment, cells were reseeded in the absence of drugs, to assess recovery. FLO‐1 cells with reduced MxA expression were significantly more sensitive to 5‐FU (Figure 3B). In fact, colony re‐growth was even decreased in untreated cells by 30% (*p* = 0.0033). This effect was enhanced following 5‐FU treatment at all concentrations tested (10–30 μM) (*p* < 0.001, *p* = 0.002, *p* = 0.0013, *p* = 0.034, and *p* = 0.0024, respectively). The levels of active caspase‐3 were also assessed. Interruption of MxA expression caused a significant increase in caspase‐3 activation in both untreated cells (*p* < 0.001) and cells treated with 5‐FU (10 and 20 μM) (*p* = 0.002 and *p* = 0.001, respectively; Figure 3C i and ii).

The response of FLO‐1 cells to a second drug, oxaliplatin (0.5–2 μM), was also evaluated. Again, depletion of MxA had a significant impact on the ability of cells to recover from oxaliplatin (Figure 3D) (*p* = 0.0017 at 0.75 μM and *p* < 0.001 at all other concentrations) and led to a fivefold increase in the percentage of caspase‐positive cells (Figure 3E i and ii) in untreated and oxaliplatin‐treated FLO‐1 cells (*p* < 0.001).

Therefore, interruption of MxA expression in FLO‐1 cells is associated with increased susceptibility to cell death in untreated and drug (5‐FU and oxaliplatin)‐treated cells. It is possible that classical apoptosis plays a major role in this death, as caspase‐3 activation is also significantly increased by the depletion of MxA in this cell line.

### 3.4 Knockout of MxA increases the sensitivity of OE21 cells to 5‐fluorouracil and oxaliplatin but apoptosis is decreased

**FIGURE 4:**
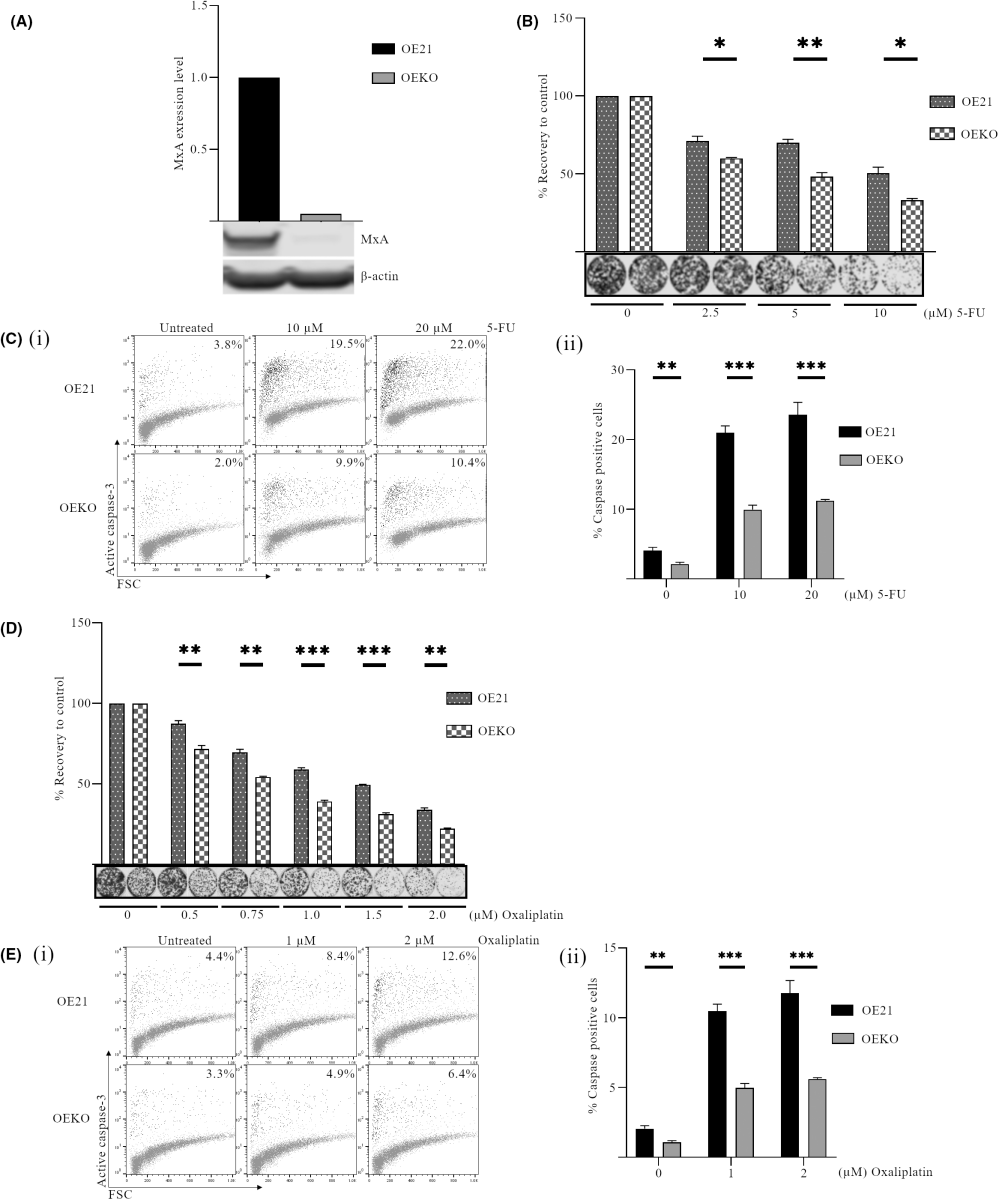
CRISPR knockout of MxA increases sensitivity of OE21 cells to 5‐fluorouracil (5‐FU) and oxaliplatin but active caspase‐3 is decreased. MxA expression was permanently knocked out via CRISPR. This new OE21‐*MX1* ^KO^ (OEKO) cell line and parental OE21 cells were treated for 24 h with either 5‐FU (2.5–20 μM) or oxaliplatin (0.5–2.0 μM). (A) MxA Knockout was confirmed by western blot. β‐Actin was used as a loading control and all bands were quantified using the Odyssey infrared imaging system. (B and D) Following relevant drug treatment, OE21 and OE21‐*MX1* ^KO^ (OEKO) cells were reseeded in drug‐free medium to examine their ability to recover and form colonies. Colonies were stained using Rapi‐diff and quantified using the Odyssey infrared imaging system. Triplicate data are presented as integrated intensity ± SEM. (C and E) Levels of active caspase‐3 were assessed by flow cytometry. Representative dot plots are shown (i) with mean percentage caspase‐positive cells for three independent experiments graphed in (ii) ± SEM (*n* = 3). (*p < 0.05, **p < 0.01, ***p < 0.001).

To evaluate the effect of depleting MxA in an additional expressing cell line, we had to employ CRISPR to knockout the gene, as we were unsuccessful at achieving a reproducible knockdown with siRNA in OE21 cells. It is clear from the Broad cell line database (https://depmap.org/portal/interactive/) that OE21 cells have a very high level of MxA transcript (see cell line comparison in Figure S4), which may be due to genetic or epigenetic alterations in this cell line. Therefore, we employed CRISPR‐Cas9 technology to permanently knockout the gene for MxA–*MX1*, creating a new cell line; OE21‐*MX1* ^*KO*^ (OEKO). The knockout was confirmed by western blot (Figure 4A) and PCR of the target exon 10 (Figure S5).

OE21 parental and MxA knockout (OEKO) cells were compared for their response to treatment with 5‐FU (2.5–10 μM) for 24 h. OEKO cells were significantly more sensitive to all concentrations of 5‐FU (2.5, 5, and 10 μM) (*p* = 0.023, *p* = 0.0031, *p* = 0.011) (Figure 4B), although the extent of sensitization was not as significant as that seen in FLO‐1 cells. In contrast to the FLO‐1 MxA‐KD cells, OEKO showed reduced caspase‐3 induction in untreated cells (*p* = 0.0025) and 5‐FU‐treated cells (10 and 20 μM) (*p* < 0.001), when compared to parental OE21 cells (Figure 4C i and ii).

OEKO cells were also more sensitive to oxaliplatin (0.5–2 μM) (Figure 4D) (*p* = 0.0054, *p* = 0.0017 at 0.5 and 0.75 μM, *p* < 0.001 at all other concentrations). In addition, there was a twofold decrease in the percentage of caspase‐positive cells in the MxA KO cell line in untreated (*p* < 0.001) and oxaliplatin‐treated cells (1 and 2 μM) (*p* = 0.0033, *p* < 0.001; Figure 4E i and ii). This reduction in caspase‐3‐dependent apoptosis is consistent with both chemotherapeutic drugs in OEKO cells.

Therefore, loss of MxA expression in OE21 cells is associated with reduced survival, irrespective of the chemotherapeutic tested. This is consistent with FLO‐1 cells. However, in contrast to FLO‐1 cells, there is no correlation with apoptosis in OE21 cells. In fact, apoptosis is decreased in cells that are more drug‐sensitive. While induction of apoptosis may contribute to the dramatic sensitization observed in the FLO‐1 cells, it is not required for a chemo‐sensitizing effect in OE21 cells.

Finally, in the fifth cell line (KYSE270) there were very low levels of MxA detectable by western blot (Figure 1A). We depleted this expression with siRNA (Figure S6A), and there was no significant effect on drug sensitivity or active caspase‐3 levels (Figure S6B,C). This suggests that there may be a threshold of expression at which inhibition of MxA would be useful.

In summary, in esophageal cancer cells that have naturally lost MxA expression, expression is not simply restored with IFN‐α alone. Artificial over‐expression can either marginally increase survival (as in KYSE450 cells) or have no effect (KYSE140). When we deplete MxA from both FLO‐1 and OE21 cells, we see a consistent decrease in survival, indicating that MxA inhibition may be a useful strategy for sensitization in esophageal cancer cell lines that express MxA. However, the inconsistencies in the activation of caspase‐3 suggest that although increased sensitivity can be significantly augmented by caspase‐3‐mediated apoptosis, enhancement of sensitivity is not reliant on apoptosis induction.

## 4 DISCUSSION

This study has evaluated whether there is a relationship between MxA expression, apoptosis induction, and sensitivity of esophageal cancer cells to chemotherapeutic drugs. While all these parameters can be affected through manipulation of MxA, there is no simple correlative relationship from this data. Instead, this data suggests a complex relationship, which is likely to be dependent upon other signaling pathways present in a particular cell line.

Previous studies had indicated that expression of MxA might sensitize cells to death‐inducing stimuli.^14^, ^17^, ^24^, ^26^, ^27^, ^28^ We therefore explored this possibility in two MxA‐null, apoptosis incompetent cell lines that could resist drug treatments for significantly longer than other cell lines. Expression of MxA did not restore apoptosis, in fact, the recovery of KYSE450 cells following drug treatment was slightly improved by over‐expression of MxA. The lack of endogenous expression of MxA in these two cell lines is not reflective of the proteomics study in esophageal squamous cell carcinoma, which indicated that upregulation of MxA is common.^21^ However, it is important to note that the patient samples in the proteomics study will reflect more localized tumors, as metastatic stage disease is usually inoperable. It is possible that these two cell lines reflect the scenario reported in prostate cancer, where MxA expression is lost as the disease progresses and the more metastatic models do not express MxA.^30^ In the prostate cancer models, low MxA expression was reported to promote other features such as proliferation and migration.^17^ It is possible that the improved clonogenic re‐growth in the KYSE450 cells was not related to drug resistance but to other features enabling more robust re‐growth following treatment. Interestingly, KYSE450 cells were recently predicted to fall into a “high risk” subtype of esophageal cancer, based on consensus clustering of proteomics data from esophageal tumors.^21^

We also considered the possibility that MxA might only be fully functional in the presence of other IFN‐α induced proteins (*ISGs*) that it co‐operates with. In addition, MxA can be post‐translationally modified by other ISG's^31^ and this might affect its function.

Treatment with IFN‐α did increase drug sensitivity in the esophageal cancer cell lines, which is consistent with many other cell line models,^32^ however, this was unaffected by the presence of MxA. It is interesting to note that both KYSE450 and KYSE140 cells were unable to induce MxA expression in response to interferon stimulation (Figure 2D and Figure S3D). This is unusual as MxA expression is considered one of the most reliable markers of IFN activity.^33^ Neither of these cell lines has a frameshift or stop codon in the coding sequence of MxA (https://depmap.org/portal/). Interestingly, the proteomic study on esophageal cancer also noted that the immune response genes that they identified as differentially expressed (*MX1*, *IDO1*, *IFIT1*, and *IFIT3*) did not correspond to alterations in published genomic data.^21^ This suggests there may be epigenetic, epi‐transcriptomic, or post‐translational regulation of MxA expression. Epigenetic silencing of *MX1* has been reported in head and neck cancer and this was re‐activated by the demethylating agent 5‐aza‐2′‐deoxycytidine.^34^ The absence of MxA in two of five esophageal cell lines in this study suggests that there may have been a selective pressure to deplete MxA during the development or progression of the original cancers.

We also evaluated the effects of MxA depletion in apoptosis‐competent cell lines with constitutive or endogenous MxA expression (FLO‐1, OE21, and KYSE270 cells). In the FLO‐1 cells, depletion of MxA expression led to a 3‐fold increase in sensitivity to two chemotherapeutic agents and extensive induction of apoptosis. This would be a highly encouraging sensitization approach if it could be replicated in other cancer cell lines. OE21 cells with MxA knocked out also showed increased sensitivity to chemotherapeutic agents, but to a lesser extent, and in contrast to FLO‐1 cells, caspase‐3‐mediated apoptosis was decreased. This suggests another type of cell death pathway must be involved in this sensitization. KYSE270 cells had very low levels of MxA, and their drug sensitivity was unaffected by its depletion. We do not know why the apoptotic response is so different in the OE21 and FLO‐1 cells.

In the context of other studies examining the effect of interruption of MxA expression on cell viability, the data with OE21 cells is analogous to observations in DU145 prostate cancer cells. When MxA was depleted in DU145 cells they also reported less apoptosis in response to a chemotherapeutic agent (docetaxel), but overall recovery was not assessed.^17^ Collectively, these data indicate that depletion of MxA in endogenously expressing cell lines can have an impact on apoptosis, but there must be other co‐operating interactions that influence the balance of cell death pathways and the overall impact on cell survival.

Limitations of this study include differences in the origin of the original cancers, OE21 cells are derived from a squamous cell carcinoma, whereas FLO‐1 is adenocarcinoma. In addition, depletion of MxA was achieved in two different ways in these cell lines, siRNA knockdown and CRISPR knockout. It is always possible that there are unforeseen changes in cell biology associated with these manipulations. Lastly, an unfortunate limitation of the study is that the mechanism by which MxA can influence cell death/survival remains unknown.

Attempts to define this mechanism were conducted in another study over‐expressing human MxA in Swiss mouse 3T3 cells.^26^ MxA expression accelerated cell death through at least two distinct pathways. The C‐terminal of MxA could promote drug‐induced cell death, while both N‐ and C‐terminal domains were required for cell death induced by influenza infection which included both caspase‐dependent and caspase‐independent cell death.^26^ Induction of cell death by over‐expression of MxA is not consistent with our findings in KYSE450 and KYSE140 cell lines. It is however consistent with data on the LNCaP prostate cancer cell line, where overexpression of MxA induced extensive apoptosis.^17^ As this effect on apoptosis was also inconsistent with other prostate cell lines in the same study, the authors speculated that it might relate to the presence of WTp53 in LNCaP cells. It is not clear if p53 expression is relevant to MxA activity, but it is noteworthy that all the cell lines included in our current study also have alterations in p53 (https://depmap.org/portal/ccle/).

Therefore, the impact that MxA has on apoptosis appears to be cell line dependent. The effects on each cell line, in the current study, were reproducible with two different classes of chemotherapeutic agents. In cancer cells that expressed MxA, a reduction in expression can make them more susceptible to cell death (apoptosis or other) induced by a cytotoxic agent. Strategies to target MxA for therapeutic effect would benefit from prior knowledge of its expression in a tumor. In addition, if we knew how inhibition of MxA in FLO‐1 cells achieved such a significant cytotoxic effect, this could support the design of a more targeted and effective therapeutic approach in the future.

## AUTHOR CONTRIBUTIONS

**R. M. Hayes:** Conceptualization (supporting); data curation (lead); formal analysis (lead); investigation (equal); methodology (supporting); project administration (supporting); software (equal); validation (lead); visualization (lead); writing – original draft (lead). **T. R. O'Donovan:** Conceptualization (equal); data curation (supporting); formal analysis (supporting); funding acquisition (equal); investigation (equal); methodology (equal); project administration (equal); resources (equal); software (equal); supervision (equal); validation (supporting); visualization (supporting). **S. L. McKenna:** Conceptualization (equal); data curation (supporting); formal analysis (supporting); funding acquisition (equal); investigation (supporting); methodology (supporting); project administration (equal); resources (equal); software (supporting); supervision (equal); validation (supporting); visualization (supporting).

## CONFLICT OF INTEREST STATEMENT

All authors declare no conflicts of interest.

## Supporting information


Figure S1.
Figure S2.
Figure S3.
Figure S4.
Figure S5.
Figure S6.

## Data Availability

All experimental data is included within this manuscript or supplementary data. All links to publicly available databases that were used in this study are provided within this manuscript.
