# Supplementary figures and images for: Expression of MxA in esophageal cancer cell lines can influence sensitivity to chemotherapeutic agents but this does not require apoptosis
Source: Cancer Med. 2024 Sep 16;13(17):e70173. doi: 10.1002/cam4.70173 (PMC11405456; doi:10.1002/cam4.70173)

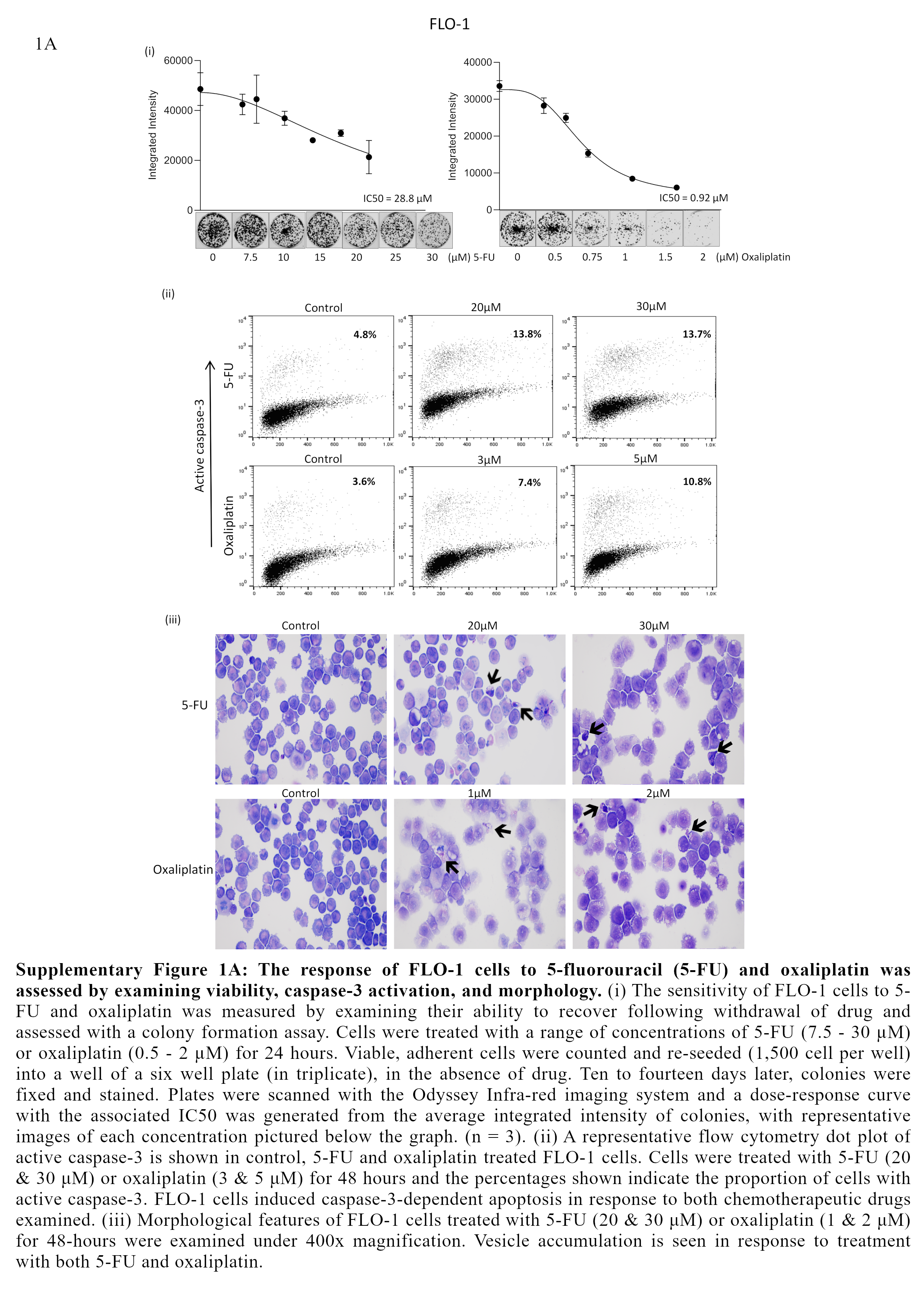

Supplement: Supplementary file 1 — Figure S1. Figure S2. Figure S3. Figure S4. Figure S5. Figure S6. [file CAM4-13-e70173-s001.zip › tiff_supplementary_figure_1A.TIFF]

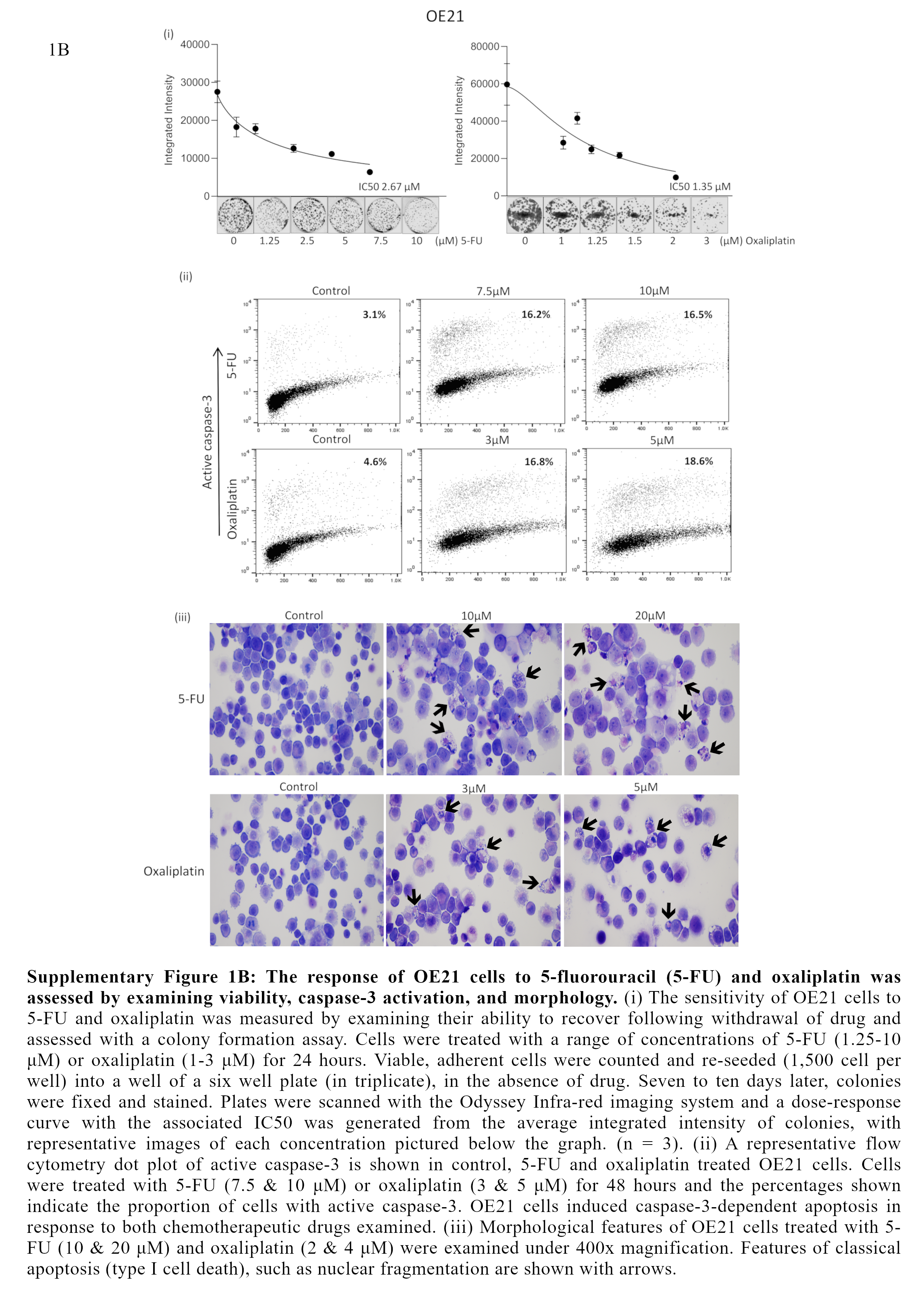

Supplement: Supplementary file 1 — Figure S1. Figure S2. Figure S3. Figure S4. Figure S5. Figure S6. [file CAM4-13-e70173-s001.zip › tiff_supplementary_figure_1B.TIFF]

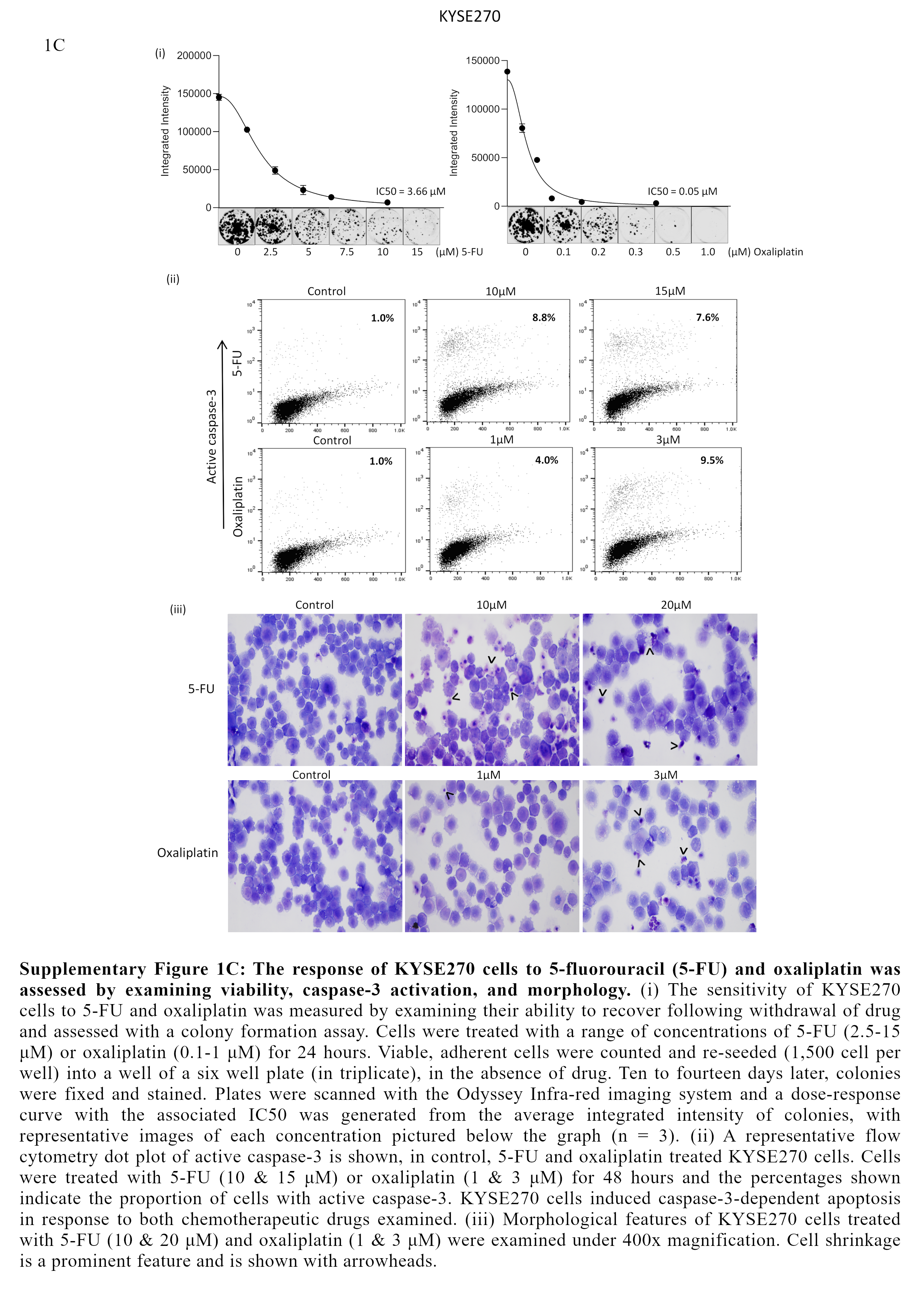

Supplement: Supplementary file 1 — Figure S1. Figure S2. Figure S3. Figure S4. Figure S5. Figure S6. [file CAM4-13-e70173-s001.zip › tiff_supplementary_figure_1C.TIFF]

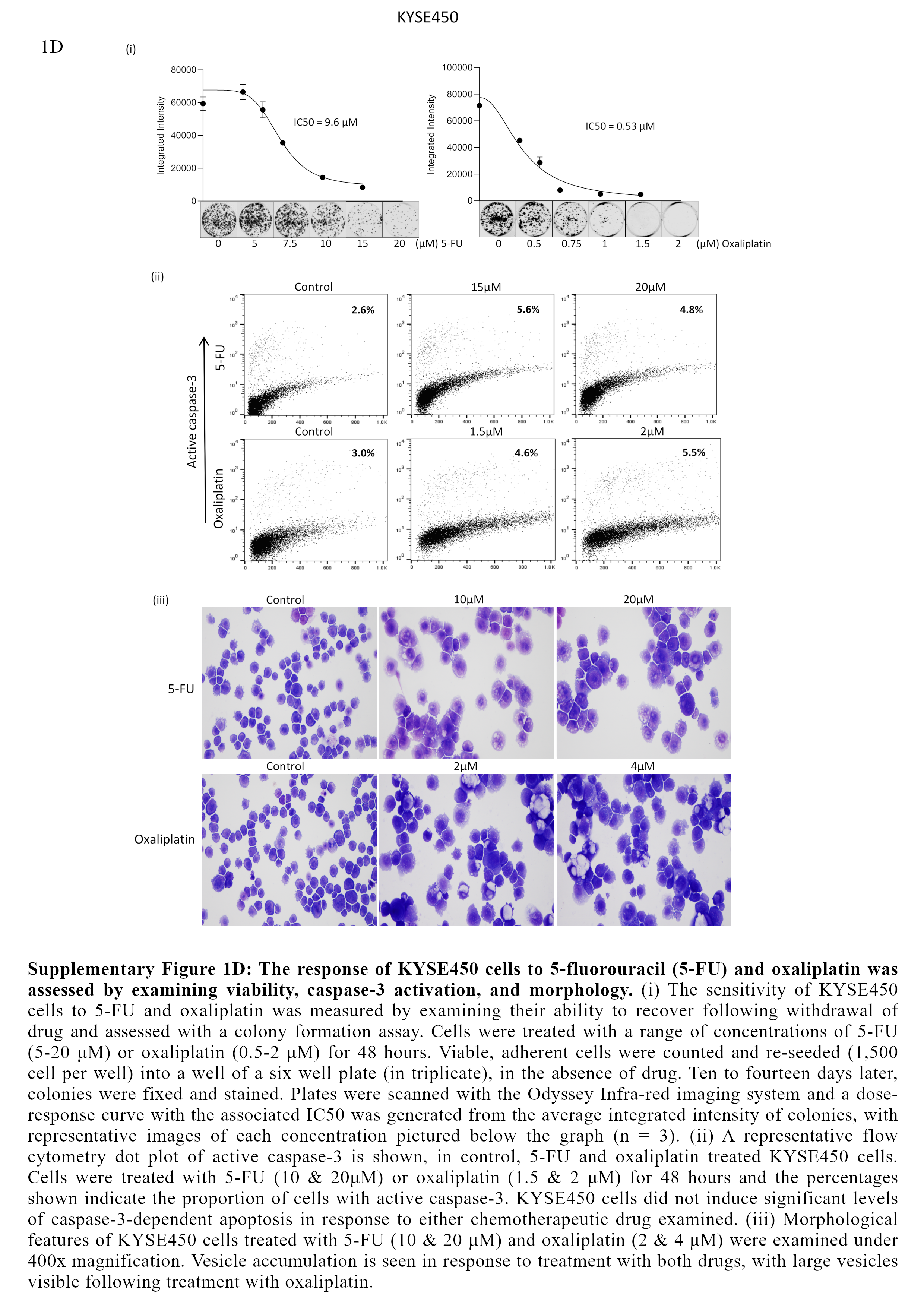

Supplement: Supplementary file 1 — Figure S1. Figure S2. Figure S3. Figure S4. Figure S5. Figure S6. [file CAM4-13-e70173-s001.zip › tiff_supplementary_figure_1D.TIFF]

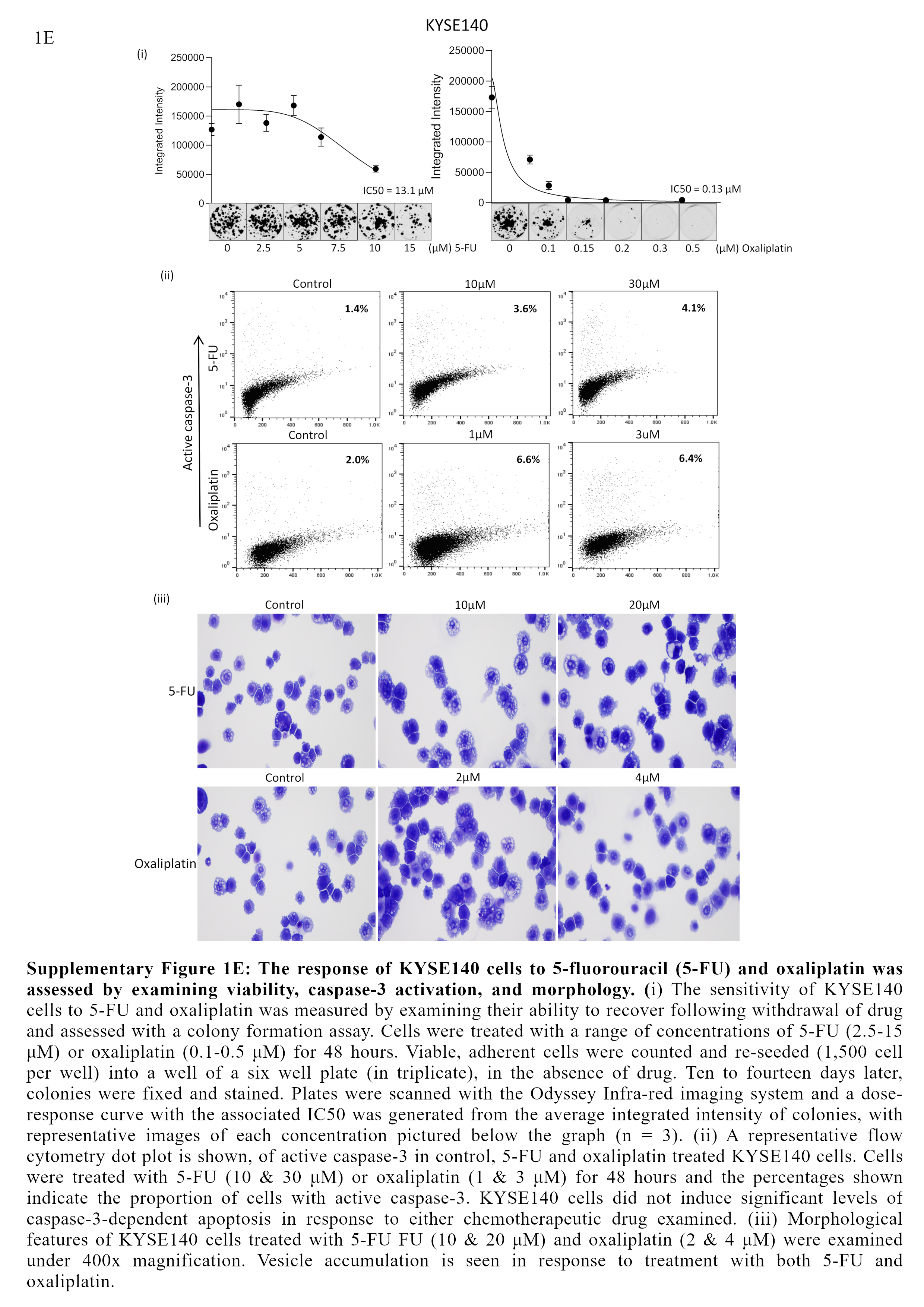

Supplement: Supplementary file 1 — Figure S1. Figure S2. Figure S3. Figure S4. Figure S5. Figure S6. [file CAM4-13-e70173-s001.zip › tiff_supplementary_figure_1E.TIFF]

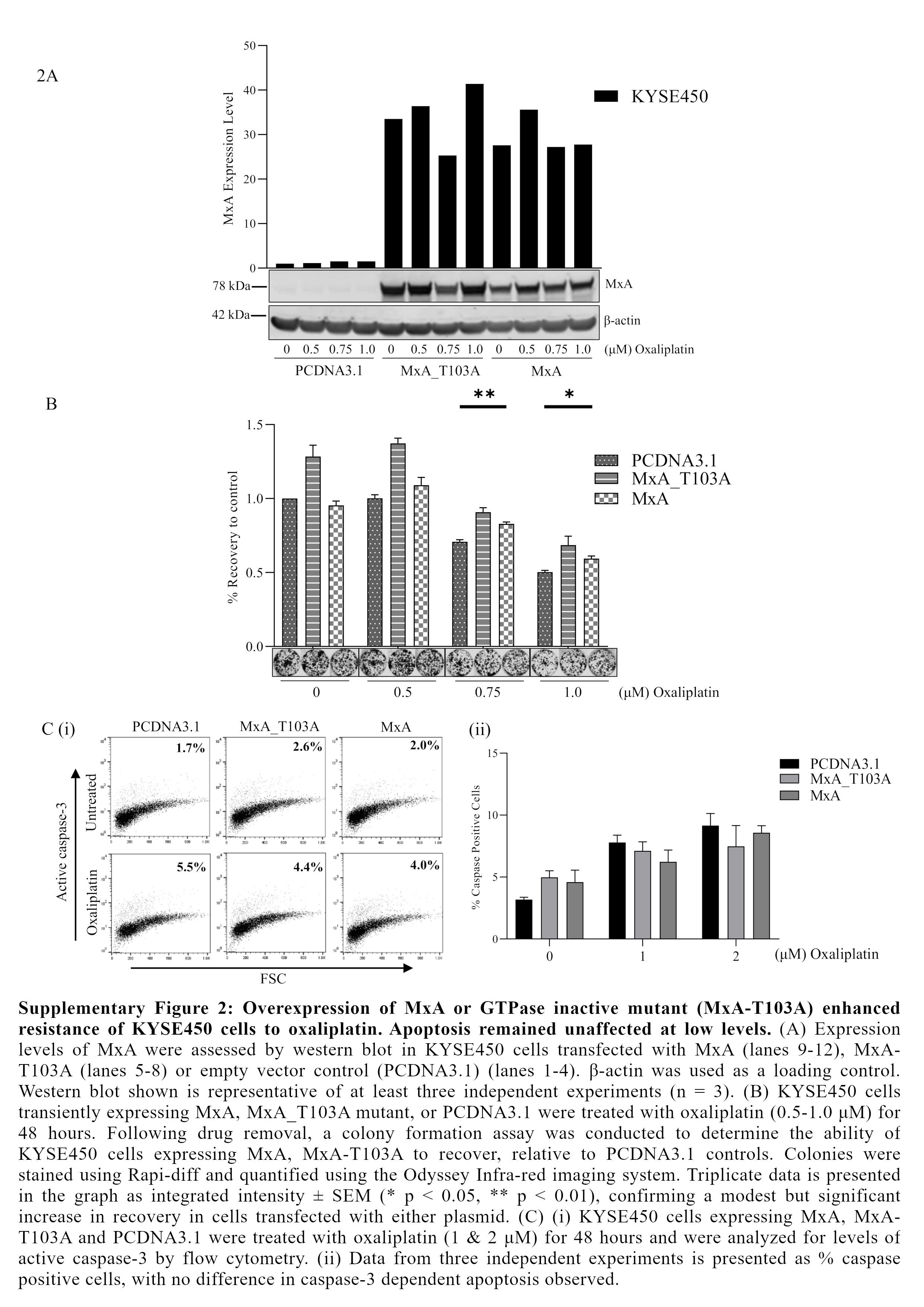

Supplement: Supplementary file 1 — Figure S1. Figure S2. Figure S3. Figure S4. Figure S5. Figure S6. [file CAM4-13-e70173-s001.zip › tiff_supplementary_figure_2.TIFF]

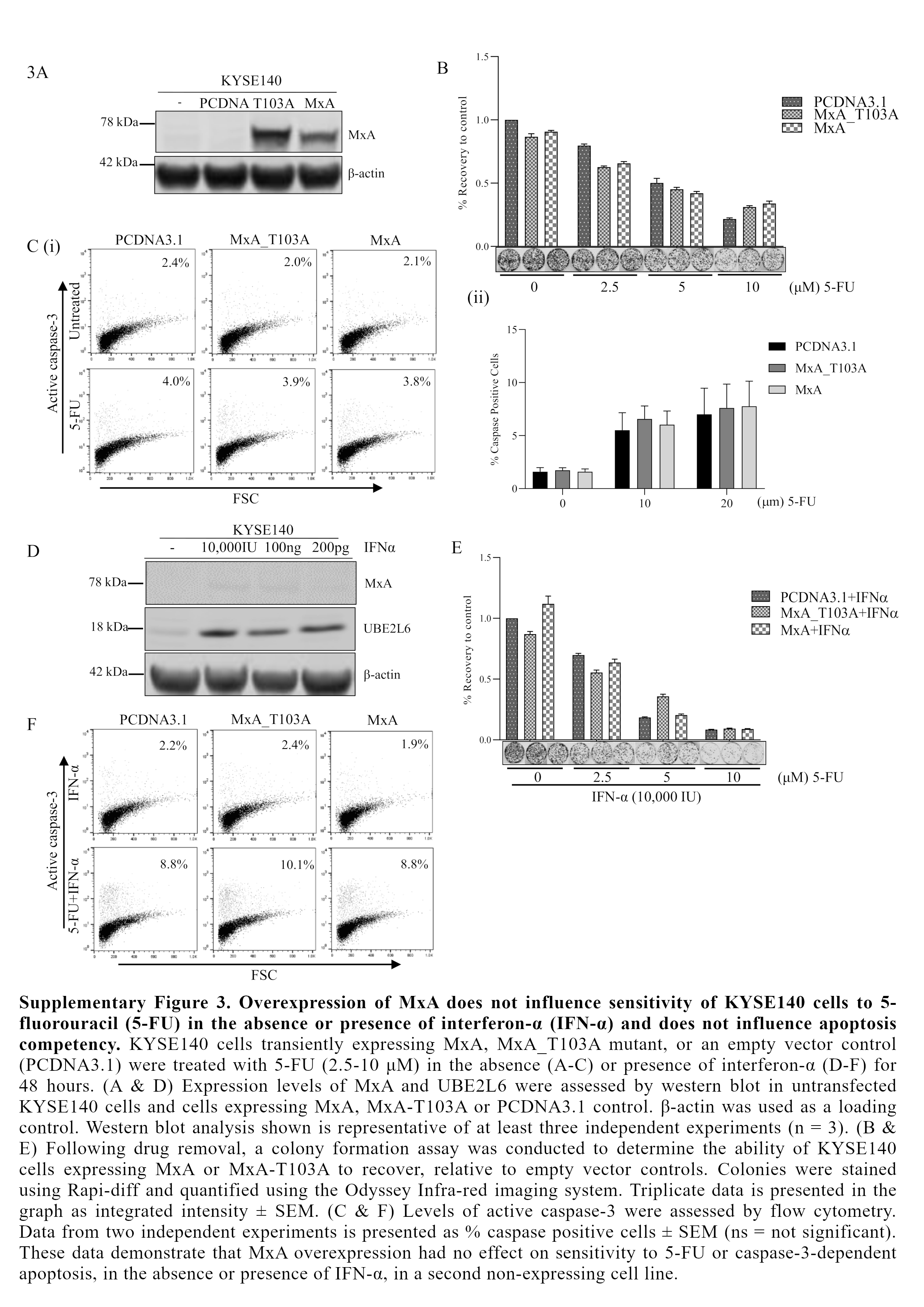

Supplement: Supplementary file 1 — Figure S1. Figure S2. Figure S3. Figure S4. Figure S5. Figure S6. [file CAM4-13-e70173-s001.zip › tiff_supplementary_figure_3.tiff]

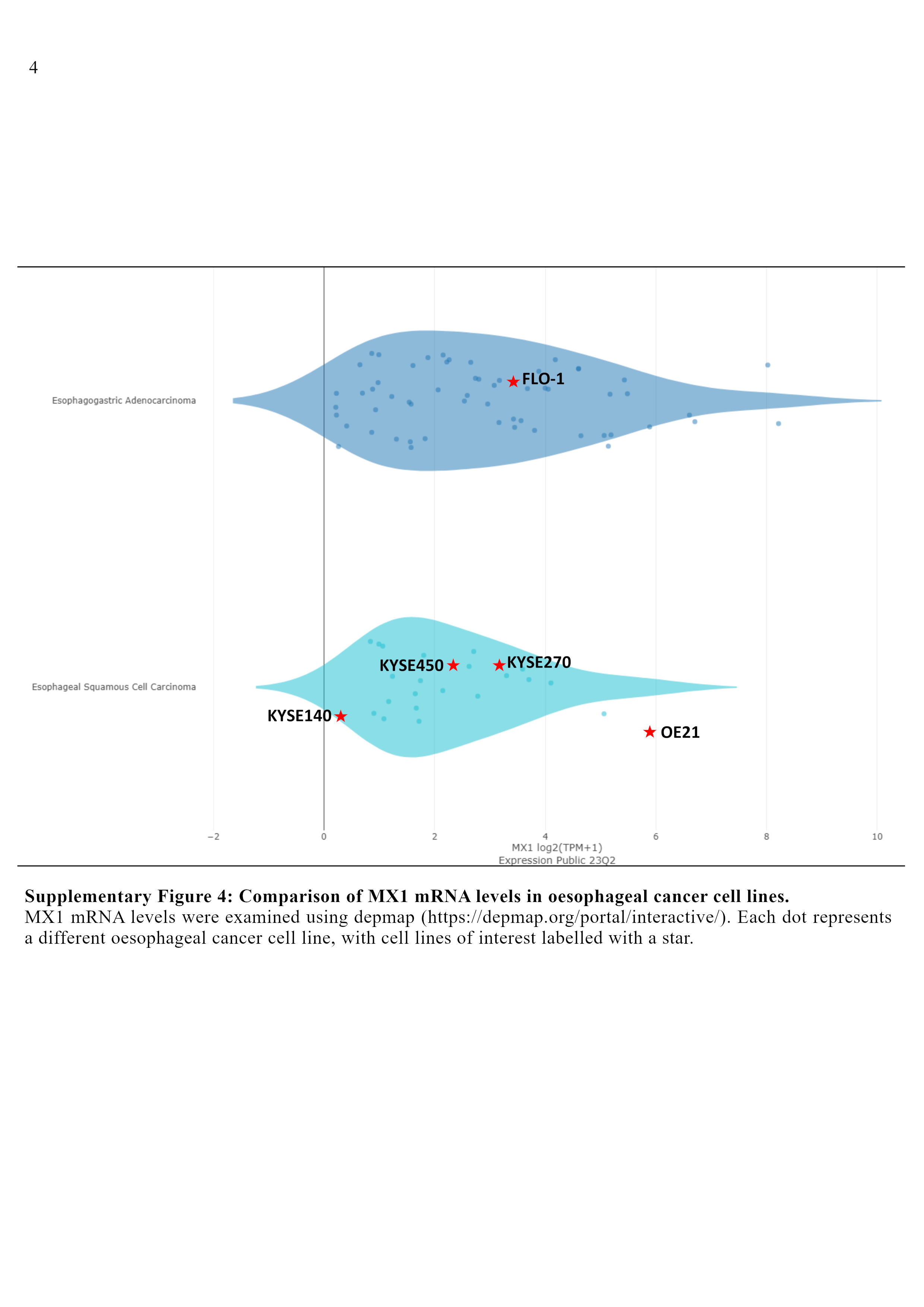

Supplement: Supplementary file 1 — Figure S1. Figure S2. Figure S3. Figure S4. Figure S5. Figure S6. [file CAM4-13-e70173-s001.zip › tiff_supplementary_figure_4.tiff]

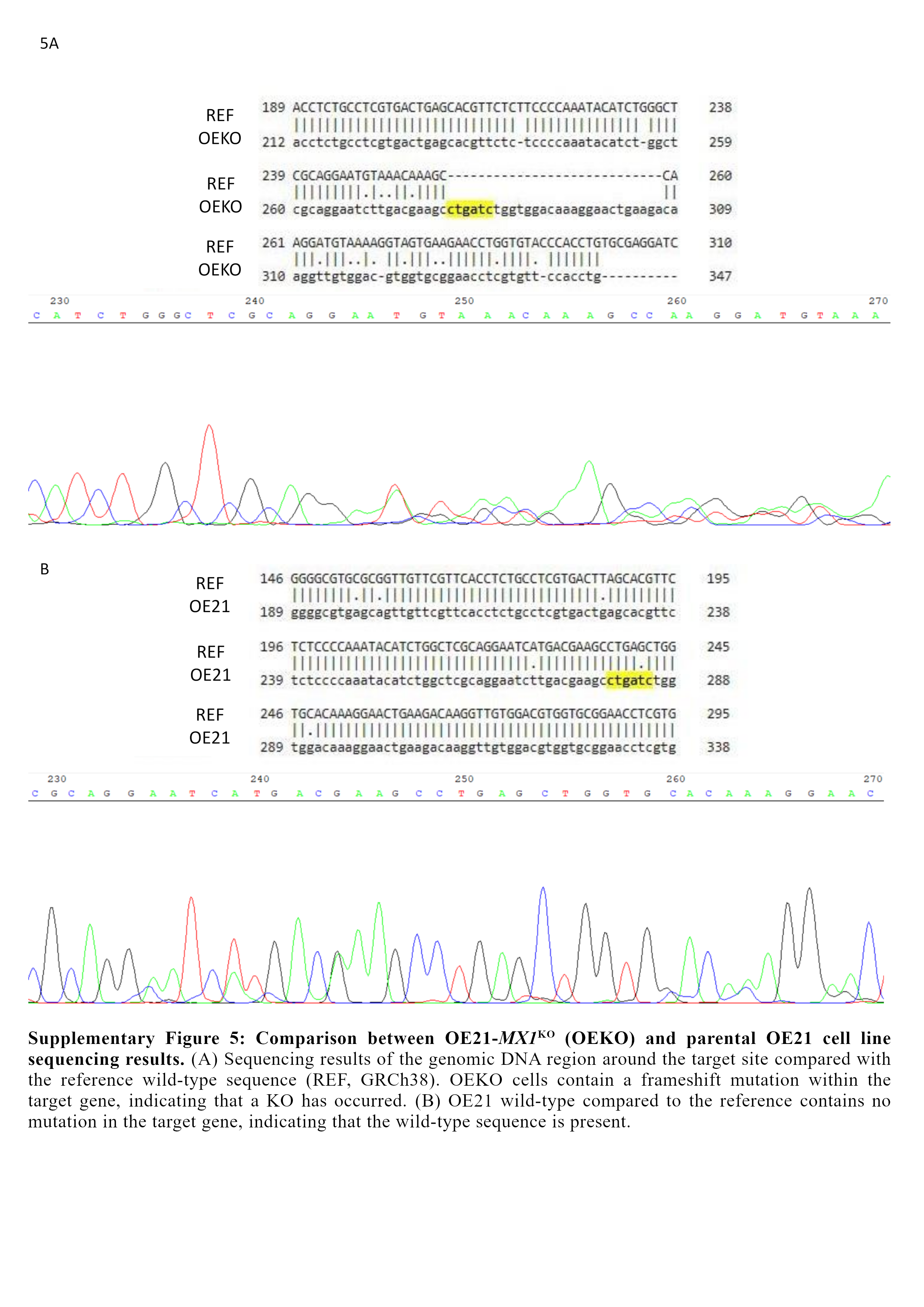

Supplement: Supplementary file 1 — Figure S1. Figure S2. Figure S3. Figure S4. Figure S5. Figure S6. [file CAM4-13-e70173-s001.zip › tiff_supplementary_figure_5.TIFF]

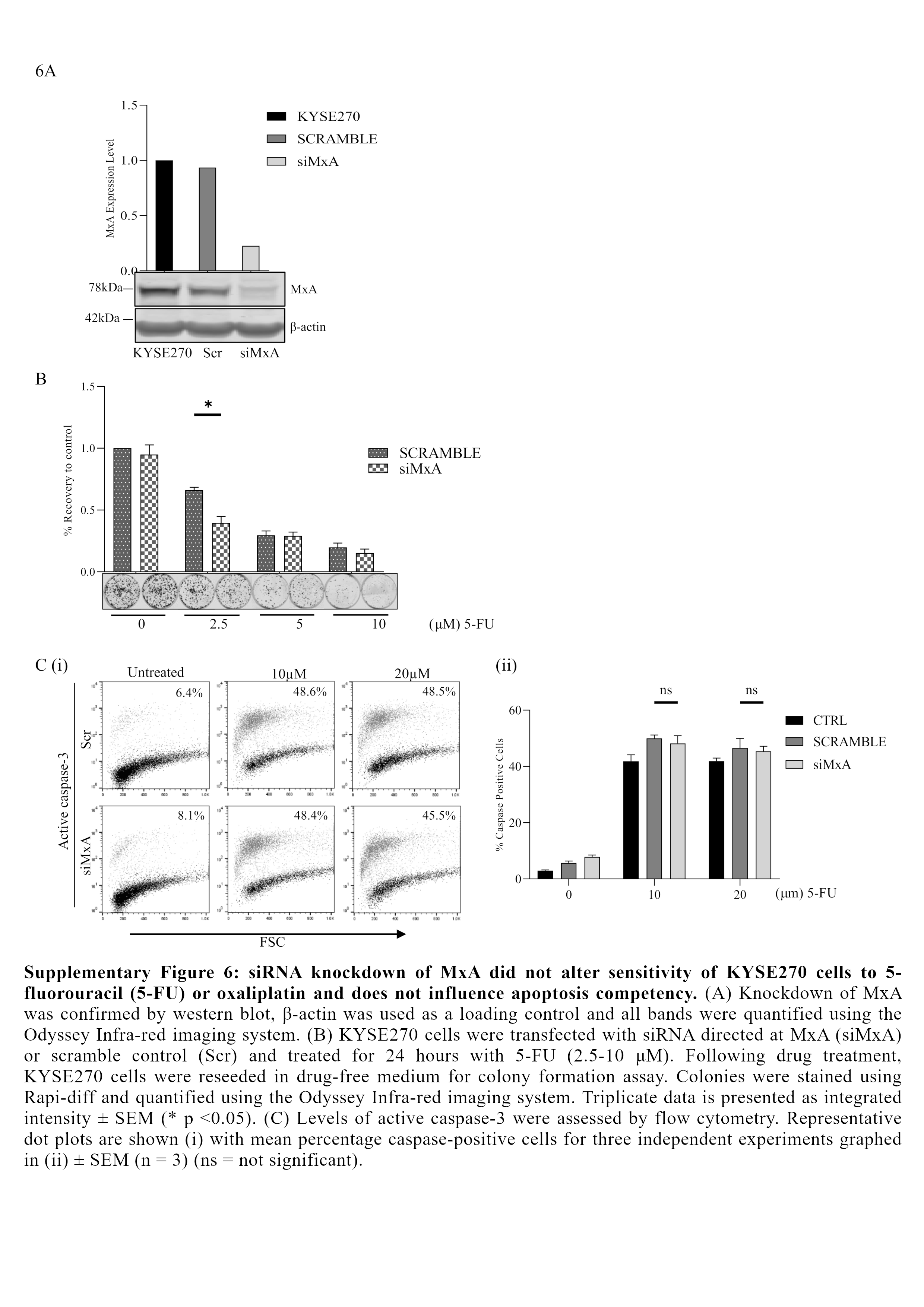

Supplement: Supplementary file 1 — Figure S1. Figure S2. Figure S3. Figure S4. Figure S5. Figure S6. [file CAM4-13-e70173-s001.zip › tiff_supplementary_figure_6.tiff]
